# GAI hallucinations and user dependence: cognitive-affective responses and individual differences

**DOI:** 10.3389/fpsyg.2026.1939367

**Published:** 2026-10-09

**Authors:** Rongqin Liu, Hui Jiang, Fangying Pang

**Affiliations:** College of Business, Nanning University, Nanning, China

**Keywords:** cognitive dissonance, GAI dependence, GAI trust, generative artificial intelligence hallucinations, self-efficacy

## Abstract

Generative artificial intelligence (GAI) hallucinations raise questions about how users respond to unreliable outputs from a technology on which they depend. Drawing on the cognitive–affective components of the Interaction of Person–Affect–Cognition–Execution (I-PACE) framework, this study examines the roles of GAI trust, cognitive dissonance, perceived controllability, and frustration in the relationship between perceived hallucinations and GAI dependence, with GAI literacy and self-efficacy as individual-difference moderators. A between-subjects experiment involved 546 participants who viewed the same GAI dialogue labelled as either containing fabricated information or verified as accurate. Participants in the former condition reported lower trust and perceived controllability, greater cognitive dissonance and frustration, and lower dependence scores. In the parallel model, the indirect associations through trust, controllability, and frustration were negative, whereas the indirect association through cognitive dissonance was positive. Higher GAI literacy weakened the positive controllability–dependence association. The remaining moderation hypotheses were not supported under the directional and multiplicity-adjusted criteria. The findings connect users’ evaluations of unreliable GAI responses with the difficulties they report in regulating their use. They underscore the importance of examining dependence alongside reliability judgements, cognitive conflict, and emotional reactions when assessing the human consequences of GAI hallucinations.

## 1 Introduction

Generative artificial intelligence (GAI) is used for information seeking, writing, and academic or professional assistance. These uses can make the technology a valued resource, but the amount of use does not by itself explain whether that relationship becomes problematic. Research on conversational AI and educational use has examined difficulties regulating engagement, escape-oriented motives, and adverse consequences alongside ordinary instrumental use (Hu et al., 2023; Huang et al., 2024; Zhang et al., 2024). In this study, GAI dependence concerns this symptom-oriented pattern: using GAI to escape personal problems, difficulty reducing use, discomfort when access is restricted, and interference with academic or occupational functioning. This definition directs attention to the quality and consequences of the user–technology relationship rather than equating dependence with frequent use or efficient task delegation.

GAI hallucinations introduce a particular challenge to that relationship. Outputs may appear coherent while containing fabricated or unsupported information, complicating users’ judgements about what to trust (Huang et al., 2025; Sun et al., 2024). Knowing that a system can make mistakes differs from judging a particular response to be erroneous. Once an answer is understood as containing a hallucination, a user may question the system, reconsider the decision to rely on it, assess whether the problem is manageable, and experience frustration. These reactions concern both the answer and the user’s relationship with GAI. Whether they have similar or competing associations with dependence is an empirical question.

The distinction between reliance and dependence is central to this question. Trust may encourage accepting assistance, but accepting assistance is not equivalent to difficulty limiting its use (Lee and See, 2004). Likewise, continuing a conversation can serve information checking rather than emotional reliance. Prior work has examined hallucination-related risks and continued use (Lim et al., 2025), cognitive dissonance and intermittent discontinuance (Zhou and Zhang, 2025), and cognitive–emotional pathways to GAI reuse (Liu et al., 2026). Research also examines frustration, coping, and continued engagement with GAI (Satoto et al., 2025). These contributions establish that post-use reactions are not limited to straightforward rejection. They leave a distinct question about how evaluations of an unreliable response relate to symptoms of problematic use, rather than merely to intentions to continue using a tool.

The Interaction of Person–Affect–Cognition–Execution (I-PACE) framework connects personal characteristics, technology-related expectations, affective responses, and problematic use (Brand et al., 2016, 2019, 2025). It provides a basis for examining why unfavourable experiences with GAI may have different implications for users’ dependence. Trust concerns the credibility of the support that GAI provides; cognitive dissonance reflects conflict about relying on that support; perceived controllability concerns whether problems in its responses can be managed; and frustration captures the emotional cost of an unsatisfactory response. GAI literacy and self-efficacy may shape the importance of these evaluations by influencing how users perceive their resources for working with the technology. Building on this theoretical foundation, the present study seeks to address the following research questions:

*RQ1*: How are the cognitive and emotional responses associated with perceived GAI hallucinations related to GAI dependence?

*RQ2*: How do GAI literacy and self-efficacy moderate the relationships between these cognitive–affective responses and GAI dependence?

The study connects research on GAI hallucinations with research on problematic technology use. It examines dependence alongside four distinct aspects of the user experience: confidence in the system, conflict about relying on it, appraisals of controllability, and frustration. It also considers whether application-oriented literacy and task-related self-efficacy help explain individual differences in these relationships. Together, these questions focus attention on how evaluations of unreliable outputs relate to the difficulties users experience in regulating their engagement with GAI.

## 2 Literature review

### 2.1 GAI hallucinations and user responses

Hallucinations refer to plausible GAI outputs that are factually incorrect, fabricated, or unsupported by relevant evidence. Technical research examines their origins, classification, detection, and mitigation (Huang et al., 2025; Sun et al., 2024). For users, a central difficulty is that linguistic fluency and factual accuracy need not coincide. A response can seem credible until it conflicts with other information or is challenged by an external assessment. Hallucination-related experiences therefore include encountering inaccurate content, suspecting an error, independently verifying it, and being informed that an answer is erroneous.

User-oriented research has considered several consequences of unreliable output. Lim et al. (2025) examined hallucination concerns in a model of continued use and premium subscription intentions. Zhou and Zhang (2025) connected information hallucination and cognitive dissonance with intermittent discontinuance. More recent work examined cognitive expectations, emotion, and self-efficacy in relation to GAI reuse (Liu et al., 2026). Satoto et al. (2025) investigated a stress-coping account of frustration and continuing GAI engagement. These studies have already established a role for cognitive and affective responses in post-adoption decisions. The remaining question is how those responses relate to dependence-related difficulties, rather than whether negative experiences can ever coexist with continued use.

Users’ judgements of accuracy draw on both the response and information about its reliability. Yin et al. (2019) distinguished stated accuracy from accuracy experienced through model predictions, while He et al. (2023) examined accuracy statements and explanations in relation to human reliance. This work highlights the role of external information in evaluating unfamiliar content. A fluent answer may be questioned when it conflicts with other evidence or when its reliability is challenged by an external assessment. An important issue in understanding the psychological consequences of hallucinations is therefore how users interpret a response once they regard it as inaccurate.

Such an interpretation may extend beyond the credibility of one answer. A user who values GAI as a source of assistance may question both the tool and the wisdom of relying on it, while also considering whether its errors can be corrected. The accompanying frustration may make the tool less reassuring, even as an established need for its support remains. Examining these evaluations together provides a basis for understanding why perceptions of unreliable output may have different associations with GAI dependence.

### 2.2 GAI dependence and related forms of use

GAI dependence refers here to problematic use characterised by escape-oriented engagement, difficulty reducing use, discomfort when access is restricted, and adverse academic or occupational consequences. These features follow the dependence-related content of the instruments from which the study’s items were adapted (Andreassen et al., 2012; Hu et al., 2023). They place difficulties regulating engagement and the consequences of that engagement at the centre of the construct. The construct is examined dimensionally and does not designate a clinical diagnosis.

Several neighbouring concepts require distinction. Frequency and duration describe how much a system is used. Task reliance concerns accepting its assistance or delegating a decision. Over-reliance and automation bias concern inappropriate acceptance or insufficient monitoring of automated advice relative to its reliability and the task (Lee and See, 2004; Parasuraman and Manzey, 2010). Cognitive offloading concerns the use of external resources to reduce internal processing demands (Risko and Gilbert, 2016). None is equivalent to an inability to regulate use. A productive user may engage frequently without reporting dependence-related difficulties, while someone who doubts particular answers may still experience problems limiting engagement.

Recent measurement work reinforces this need for specificity. Li et al. (2025) developed a dedicated large language model dependence scale, illustrating that dependence requires explicit definition and measurement rather than inference from use frequency. Research on problematic conversational AI use considers social anxiety, loneliness, rumination, and mind perception (Hu et al., 2023); educational studies examine academic stress, self-efficacy, and performance expectations in problematic AI usage (Zhang et al., 2024). These strands address why users may experience a need for the technology and difficulty regulating their engagement, beyond the usefulness of any individual answer.

Relational and longer-term studies add another perspective. Laestadius et al. (2024) analysed accounts of emotional dependence and harm involving Replika, where perceived human-like responsiveness and attachment were prominent. In the revised report of a four-week randomised study, Fang et al. (2025) found no significant effects of the assigned conversation conditions on psychosocial outcomes, while heavier voluntary use was associated with poorer outcomes. Lee et al. (2025) examined knowledge workers’ reports of confidence and cognitive effort during GAI-assisted work, rather than dependence symptoms or experimentally demonstrated cognitive decline. Diel et al. (2026) synthesised heterogeneous evidence on potential mental health harms from LLM-based chatbots. Together, these studies show why anthropomorphism, attachment, offloading, and problematic use should be considered without treating them as interchangeable outcomes.

### 2.3 Interaction of person-affect-cognition-execution (I-PACE) framework

The I-PACE framework explains problematic technology use through interactions among personal characteristics, affective and cognitive responses to situational cues, and executive processes involved in regulating behaviour (Brand et al., 2016, 2019, 2025). Its cognitive–affective account emphasises the significance of technology-related expectations and coping responses for the user’s relationship with a particular activity. In the context of GAI, these ideas draw attention to how expectations of reliable assistance and responses to unsatisfactory output may be associated with the perceived need for the technology and difficulties limiting its use. I-PACE is used here because the outcome concerns dysregulated engagement and its consequences. Trust-calibration accounts explain whether reliance is appropriate to system reliability, and cognitive-offloading accounts explain the delegation of processing demands; neither, by itself, specifies difficulties reducing use or interference with functioning. These perspectives inform the interpretation of trust and reliance, whereas I-PACE supplies the organising account of person-related resources and cognitive–affective responses associated with problematic use. The present model draws on this part of I-PACE rather than testing its full longitudinal account of addiction development or its executive-control component. Cognitive dissonance and appraisal concepts specify the selected responses within that structure, rather than forming additional competing models.

GAI hallucinations challenge expectations that a seemingly coherent response provides dependable assistance. The challenge can be evaluated in several ways. Trust concerns whether the system remains credible; cognitive dissonance concerns the consistency of the user’s judgements about relying on it; and perceived controllability concerns the extent to which response problems appear manageable. Frustration reflects the emotional significance of an answer that obstructs the user’s goals. Appraisal research connects these evaluations of an event and its manageability with emotional responses and coping (Beaudry and Pinsonneault, 2005; Moors et al., 2013). These distinctions help explain why the same perceived failure may be associated with both reservations about the tool and a continuing need for its support.

The tension is particularly relevant when GAI is used to manage academic demands or personal concerns. Favourable expectations can make the technology an important source of reassurance, whereas doubts about its reliability challenge the basis for that reassurance. Users may respond by reconsidering the role of GAI or by accommodating its limitations within an existing pattern of use. The latter response can leave the perceived need for the technology intact while sustaining conflict about relying on it. The proposed model consequently considers both evaluative processes that weaken attachment to GAI and conflict-related processes that may accompany stronger dependence.

GAI literacy and self-efficacy add an individual-difference perspective to these processes. Application-oriented literacy concerns perceived proficiency in selecting, prompting, and integrating GAI tools; self-efficacy concerns confidence in completing tasks with GAI under different support conditions. The resources users believe they possess may affect how closely their dependence is tied to the reliability, manageability, or emotional quality of a particular response. These constructs therefore help specify when cognitive and affective reactions are more strongly associated with GAI dependence.

## 3 Research model and hypothesis development

Drawing on this cognitive–affective perspective, the research model links the identification of a GAI answer as hallucinated to dependence through trust, cognitive dissonance, perceived controllability, and frustration. In the experiment, this identification is communicated through the label accompanying the answer. Trust and controllability concern whether the technology remains a dependable and manageable source of support. Dissonance concerns conflict about relying on that support, while frustration reflects its emotional costs. GAI literacy and self-efficacy are proposed to moderate the relationships between these responses and dependence. Figure 1 presents the research model.

**Figure 1:**
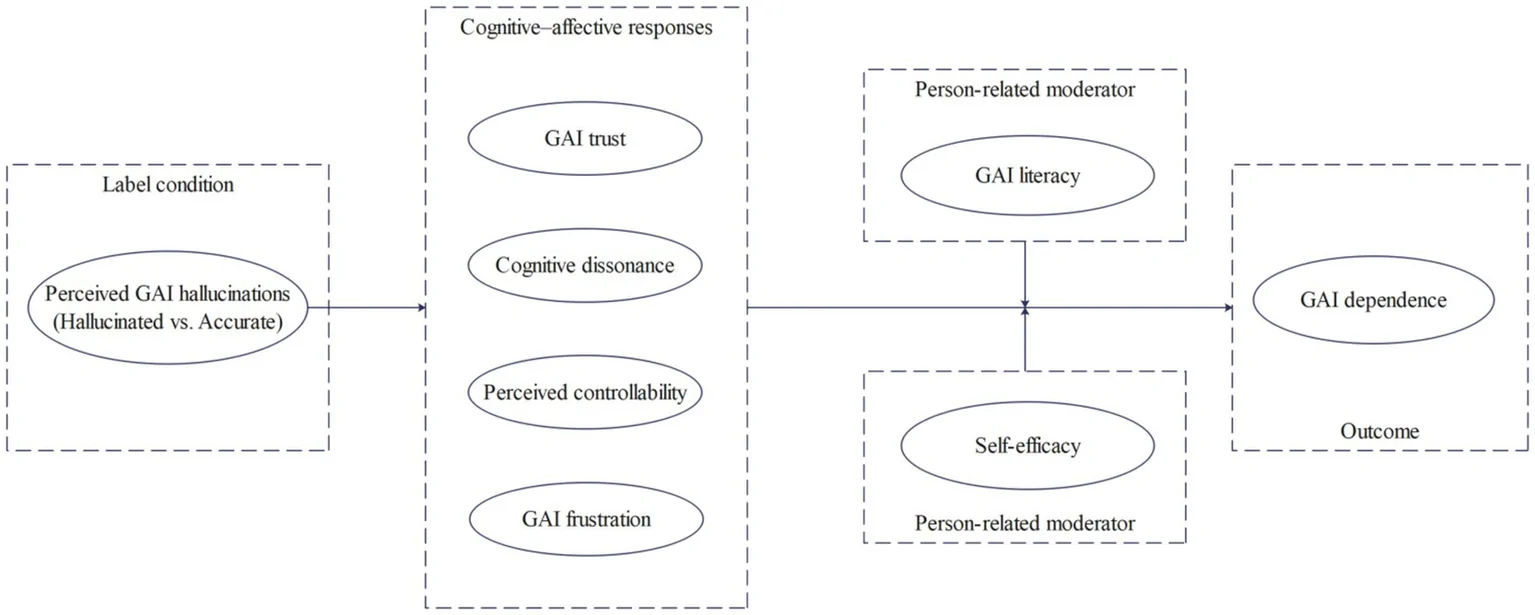
The research framework.

### 3.1 GAI trust

GAI trust reflects confidence that the system can provide reliable and capable assistance. Trust-in-automation research links such expectations to the willingness to draw on a system’s advice (Lee and See, 2004). Hallucinations challenge these expectations by revealing a discrepancy between the apparent coherence of an answer and its factual reliability. When a convincing response is understood to contain fabricated information, uncertainty extends from that response to the credibility of the system that produced it. The resulting doubt provides a reason to reconsider the system’s dependability and the confidence placed in its assistance. Trust may be particularly consequential when GAI is used to manage demanding tasks or personal concerns. Within the I-PACE framework, expectations about what a technology can provide are relevant to the cognitive and emotional processes associated with problematic use (Brand et al., 2016, 2019). We argue that confidence in GAI can sustain its perceived value as a source of reassurance and make alternatives seem less necessary. For users who already struggle to limit their engagement, these expectations may help maintain the felt need for the tool despite competing demands or adverse consequences. Identifying an answer as hallucinated challenges the reassurance that GAI is expected to provide, making it harder to sustain the belief that the tool is necessary for managing those concerns. Based on the foregoing, we propose the following hypothesis:

*H1*: Perceived GAI hallucinations have a negative indirect association with GAI dependence through GAI trust.

### 3.2 Cognitive dissonance

Cognitive dissonance arises from inconsistency among beliefs, evaluations, and behaviour, creating pressure to restore consistency (Festinger, 1957). In technology use, it can be expressed as doubt about whether adopting or relying on a system was the right decision (Marikyan et al., 2023). GAI hallucinations can create this tension when users expect dependable assistance but recognise that an answer contains false or unsupported information. The discrepancy calls into question both the system’s reliability and the user’s judgement in relying on it. Research on GAI discontinuance and ChatGPT use provides a basis for examining such conflict in AI-supported activities (Zhou and Zhang, 2025; Ren et al., 2025). For users who already regard GAI as an important resource, the conflict may be difficult to resolve because giving up the tool would also mean relinquishing a valued source of assistance. Dissonance theory allows adjustments to evaluations as well as behaviour (Festinger, 1957). Users may therefore interpret an inaccurate answer as an exception while maintaining their perceived need for GAI. Such accommodation need not eliminate doubts about relying on it. We propose that, when credibility, manageability, and frustration are considered jointly, stronger unresolved conflict can accompany a stronger perceived need for the tool and greater difficulty disengaging. In this account, dissonance concerns tension within the user–technology relationship rather than implying that recognising an error necessarily produces more use. Based on the above reasoning, we propose the following hypothesis:

*H2*: Perceived GAI hallucinations have a positive indirect association with GAI dependence through cognitive dissonance.

### 3.3 Perceived controllability

Perceived controllability concerns the extent to which GAI responses and their problems are regarded as controllable, preventable, avoidable, or correctable. Control appraisals guide users’ assessments of what can be done when a technology fails to provide the expected result (Beaudry and Pinsonneault, 2005; Song et al., 2023). A hallucinated answer can undermine confidence that appropriate inputs will produce dependable output. When plausible errors appear difficult to anticipate or correct, users have less reason to regard the system’s behaviour as manageable. Perceived hallucinations are therefore expected to be associated with lower controllability. Manageability also matters for the place that GAI occupies in users’ coping routines. Believing that inaccurate responses can be corrected may make the tool seem dependable enough to retain as a source of assistance, even when particular answers are unsatisfactory. Among users experiencing difficulties limiting GAI use, this belief may allow errors to be discounted as repairable exceptions and reduce the perceived need to reconsider the tool’s role. When responses instead appear difficult to manage, the reassurance provided by GAI becomes less secure. We consequently expect perceived controllability to be positively associated with GAI dependence. Accordingly, we propose the following hypothesis:

*H3*: Perceived GAI hallucinations have a negative indirect association with GAI dependence through perceived controllability.

### 3.4 GAI frustration

GAI frustration refers to feelings of annoyance, disturbance, and discouragement associated with an unsatisfactory response. Appraisal theory relates such emotions to events that obstruct valued goals, while service-failure research examines frustration in relation to obstacles and coping responses (Moors et al., 2013; Gelbrich, 2010). Hallucinations interfere with the expectation that GAI will provide information that helps resolve a question or complete a task. Recognising that an apparently helpful answer is unreliable may instead leave the problem unresolved and create additional uncertainty, making the encounter frustrating. Frustration can also undermine the emotional value of turning to GAI. A tool used to ease demands or obtain reassurance becomes less attractive when the encounter itself is a source of irritation. For users who turn to GAI to manage personal or academic concerns, this mismatch challenges the expectation that using it will provide relief. We therefore expect stronger frustration to be associated with a weaker sense of dependence on GAI. Based on the above reasoning, we propose the following hypothesis:

*H4*: Perceived GAI hallucinations have a negative indirect association with GAI dependence through GAI frustration.

### 3.5 GAI literacy and self-efficacy as moderators

GAI literacy encompasses competencies for working with AI technologies (Long and Magerko, 2020). The application-oriented dimension considered here concerns perceived proficiency in prompting GAI, selecting appropriate functions, solving tasks, and integrating tools with domain knowledge (Shi et al., 2025). These capabilities may shape the relationship between users’ evaluations of GAI and their dependence by affecting the range of uses and responses they believe are available to them. When users trust GAI, greater perceived application proficiency may allow that trust to extend across more activities. A trusted tool can become more central to managing everyday demands when users believe they can employ it effectively in varied settings. We therefore expect literacy to strengthen the positive trust–dependence relationship. Its role may differ when the user experiences dissonance. A broader perceived repertoire for working with GAI may make it easier to reconsider a particular approach without relying on continued engagement to defend the original use decision. Literacy is consequently expected to weaken the positive relationship between dissonance and dependence. Literacy may also limit how strongly one difficult response shapes the broader relationship with GAI. For users who perceive few application skills, a manageable answer can provide important reassurance, whereas an unmanageable or frustrating one can substantially weaken that reassurance. Users who perceive a wider range of ways to work with GAI may attach less weight to the manageability and emotional quality of one response. We therefore expect higher literacy to attenuate both the positive controllability–dependence relationship and the negative frustration–dependence relationship. Thus, we propose the following hypotheses:

*H5a*: Higher GAI literacy strengthens the positive association between GAI trust and GAI dependence.

*H5b*: Higher GAI literacy attenuates the positive association between cognitive dissonance and GAI dependence.

*H5c*: Higher GAI literacy attenuates the positive association between perceived controllability and GAI dependence.

*H5d*: Higher GAI literacy attenuates the negative association between GAI frustration and GAI dependence.

GAI self-efficacy refers to confidence in one’s ability to accomplish tasks with GAI under different levels of experience and support (Bandura, 1977; Rodríguez-Ruiz et al., 2025; Shao et al., 2025). It concerns whether users believe they can act effectively when working with the technology. Research on technological stress and service difficulties links efficacy beliefs to coping and affective responses (Chang et al., 2024; Tsarenko and Strizhakova, 2013), providing a basis for examining their role in responses to GAI hallucinations. When confidence in the system is accompanied by confidence in one’s own ability to use it, GAI may be more readily incorporated into activities through which users manage demands and seek reassurance. Self-efficacy is therefore expected to strengthen the positive relationship between trust and dependence. In the presence of cognitive dissonance, confidence may instead provide a basis for addressing the difficulty without defending the original use decision through stronger attachment to the tool. Users who believe they can adjust their approach may feel less need to rely on GAI to resolve the unease surrounding that decision. We thus expect higher self-efficacy to weaken the positive relationship between cognitive dissonance and dependence. Task confidence can similarly reduce the importance of situational reassurance. When users are uncertain about their own capabilities, dependence may be more closely tied to whether the current response appears manageable. Stronger efficacy beliefs offer an additional basis for confidence and may make this connection less pronounced. They may also help users regard a frustrating encounter as a difficulty they can address, limiting its implications for their broader attachment to GAI. Accordingly, higher self-efficacy is expected to attenuate the positive relationship between controllability and dependence and the negative relationship between frustration and dependence. Thus, we propose the following hypotheses:

*H6a*: Higher GAI self-efficacy strengthens the positive association between GAI trust and GAI dependence.

*H6b*: Higher GAI self-efficacy attenuates the positive association between cognitive dissonance and GAI dependence.

*H6c*: Higher GAI self-efficacy attenuates the positive association between perceived controllability and GAI dependence.

*H6d*: Higher GAI self-efficacy attenuates the negative association between GAI frustration and GAI dependence.

## 4 Method

### 4.1 Design, stimuli and participants

We employed a single-factor, two-condition between-subjects design to examine users’ psychological responses when the same generative AI (GAI) answer was labelled as either hallucinated or accurate. The stimulus was a static, text-based image depicting a user–GAI dialogue about the approval of a Chinese quantum-encryption protocol or standard. This technical topic was selected to limit the potential influence of participants’ prior topic knowledge. A topic-familiarity pretest involved 30 participants assigned to the two stimulus scenarios. Familiarity was assessed with the item “How familiar are you with the approval of Chinese quantum-encryption protocols or standards?”, rated from 1 (completely unfamiliar) to 7 (very familiar). The mean familiarity rating was 1.80 (SD = 0.81); 25 participants (83.3%) selected 1 or 2. The pretest addressed topic familiarity, not validation of the full experimental procedure or all measurement scales.

The dialogue content was identical across conditions, whereas the accompanying instruction differed. Participants in the accuracy-labelled condition (control group) were shown: “Please read the following response generated by generative AI (e.g., DeepSeek). This content has been verified by experts and confirmed to be accurate GAI output.” Participants in the hallucination-labelled condition were shown: “Please read the following response generated by generative AI (e.g., DeepSeek). This content has been confirmed to contain false or fabricated information and constitutes hallucinated GAI output.” The dialogue stimulus is provided in Appendix A.

The experimental factor was the accuracy-related label accompanying the answer, coded 0 for the accuracy-labelled condition and 1 for the hallucination-labelled condition. Holding the dialogue constant controlled its wording and topic while varying the information provided to participants when evaluating the answer. The design therefore manipulated externally communicated accuracy information rather than objective answer accuracy. No unlabelled condition was included. Because expert verification was mentioned only in the accuracy-labelled instruction, accuracy assurance and expert endorsement were not independently manipulated.

A total of 546 participants were recruited between October 15 and October 30, 2025, and assigned to the two conditions. The analytical sample comprised 273 participants in each condition. The experiment was conducted in a room, and participants completed the questionnaires individually on computers through Credamo. Each participant received a small gift upon completion. Demographic characteristics are presented in Table 1. Students accounted for 386 participants overall (70.7%), including 173 in the hallucination-labelled condition (63.4%) and 213 in the accuracy-labelled condition (78.0%). The recruitment total and the primary analytical sample were the same; the treatment of attention-check flags is described in Section 4.3.

**Table 1:** Participant characteristics by label condition.

Characteristic	Category	Hallucination-labelled (*n* = 273)	Accuracy-labelled (*n* = 273)
Gender	Male	144	135
	Female	129	138
Age	20 and below	63	42
	21–25	111	132
	26–30	54	48
	31–35	42	42
	Above 35	3	9
Education	High school or below	14	6
	Bachelor	199	189
	Master	38	42
	Doctor	22	36
Occupation	Student	173	213
	Government or education	24	12
	Company positions	66	39
	Freelancer	3	6
	Others	7	3
Reported GAI-use category	Never used	5	8
	1–3	43	50
	4–7	45	53
	8 and above	180	162

A sensitivity analysis conducted during manuscript revision characterised the detectable effect size for a simple, unadjusted between-condition mean comparison. Using a two-sided independent-samples *t* test, 273 participants per condition, *α* = 0.05, and target power of 0.80, the minimum detectable standardised mean difference was approximately Cohen’s *d* = 0.24. The calculation used TTestIndPower in statsmodels 0.14.6 and assumed independent observations and equal population variances. It was not an *a priori* sample-size calculation and does not establish power for mediation, interaction, or session-clustered analyses.

### 4.2 Measurements

Measurement items were adapted from previously published scales, with wording modifications for the GAI context. The questionnaire underwent a translation–back-translation procedure involving two experienced researchers. All construct-measurement items were rated on a seven-point Likert scale, from 1 (strongly disagree) to 7 (strongly agree).

GAI trust was measured using five items adapted from Jin et al. (2025), including “I believe GAI can accurately understand the specific questions I raise in my studies or work” (Cronbach’s *α* = 0.981). Cognitive dissonance was assessed using four items from Marikyan et al. (2023), such as “I wondered if I really needed to rely on this model’s response” (Cronbach’s *α* = 0.994).

Perceived controllability was measured using four items from Song et al. (2023), including “I believe these GAI responses are controllable” (Cronbach’s *α* = 0.975). Higher scores indicated greater perceived manageability of GAI responses. The construct was retained in this scoring direction and was not relabelled as behavioural loss of control. GAI frustration was measured using three items from Gelbrich (2010), including “I would feel frustrated about these GAI responses” (Cronbach’s *α* = 0.994).

GAI literacy was assessed using five items from Shi et al. (2025), including “I am able to prompt GAI tools accurately by inputting text, images, or other forms of information to complete specific tasks” (Cronbach’s *α* = 0.984). The selected items concern application-oriented proficiency rather than a direct test of hallucination detection. Self-efficacy was measured using 10 items adapted from Rodríguez-Ruiz et al. (2025) and Shao et al. (2025), such as “In general, I could complete any desired task using the GAI technology, even if there was no one around to tell me what to do as I go” (Cronbach’s *α* = 0.981).

GAI dependence was assessed using four retained items, DEPE1–DEPE4, adapted from Andreassen et al. (2012) and Hu et al. (2023) (Cronbach’s *α* = 0.954). Their content concerned escape-oriented use, difficulties reducing use, discomfort when access is restricted, and adverse academic or occupational consequences. The score represents self-reported dependence-related tendencies at the post-exposure assessment. Because this content extends beyond the immediate session, the score was not treated as a state measure of newly acquired habitual dependence. Each construct score was calculated as the mean of its designated items. The analysed item set comprised 35 items: 15 for the two pre-exposure moderators and 20 for the five post-exposure constructs. Item loadings and reliability and convergent-validity estimates are provided in Appendix B.

### 4.3 Procedure

The experiment was conducted in a room where participants had access to computers for individual questionnaire completion. Before the task began, the facilitator explained the study’s purpose and procedures, and participants provided informed consent through an online information page before participation. Participants were instructed not to interact or communicate with one another throughout the experiment.

Participants first completed measures of GAI literacy and self-efficacy. The facilitator then displayed the assigned stimulus image to the group for at least 1 min, allowing participants time to read the dialogue and its accompanying instruction. Following stimulus exposure, participants completed the post-exposure questionnaire individually through Credamo. This questionnaire assessed GAI trust, cognitive dissonance, perceived controllability, GAI frustration, and GAI dependence. Participants subsequently provided their demographic information.

An instructed-response item asked participants to select “Agree” (response option 6). An isolated failure on this item was flagged for a sensitivity analysis rather than used as an automatic exclusion from the primary sample. Records were also screened for duplicate participation codes, incomplete core-item responses, completion times below 120 s, and identical responses across all 35 construct items. The timing measure covered the post-exposure questionnaire from opening to submission and excluded the earlier baseline measures and the stimulus-display period.

The final analytical sample comprised 546 complete responses, with 273 participants in the accuracy-labelled condition and 273 in the hallucination-labelled condition. Unless otherwise specified, the primary statistical analyses used this full sample; the sensitivity subsamples are reported separately in Table 2. The median questionnaire-completion time was 6.87 min (IQR = 5.63–8.40 min; range = 2.95–18.50 min). Completion-time statistics by experimental condition are presented in Table 3.

**Table 2:** Sensitivity comparisons of indirect point estimates.

Analysis	*N*	Via TR	Via CD	Via PC	Via FR	Direct
All complete responses	546	−0.568	0.436	−0.184	−0.363	0.360
Attention-check pass only	534	−0.582	0.441	−0.193	−0.368	0.371
Prior GAI users only	533	−0.568	0.416	−0.178	−0.359	0.377
Education/occupation adjusted	546	−0.549	0.437	−0.181	−0.375	0.359

**Table 3:** Questionnaire-completion and attention-check indicators.

Sample	*N*	Passed check	Flagged only	Median minutes	IQR minutes	Range minutes
Overall	546	534/546 (97.8%)	12	6.87	5.63–8.40	2.95–18.50
Accuracy-labelled	273	268/273 (98.2%)	5	6.77	5.62–8.17	2.95–13.60
Hallucination-labelled	273	266/273 (97.4%)	7	6.98	5.67–8.50	3.38–18.50

The post-exposure questionnaire included a manipulation-check item: “Do you think the GAI-generated response you just read contains any incorrect or inaccurate information?” Responses ranged from 1 (No errors at all) to 7 (Contains serious errors). Perceived error was higher in the hallucination-labelled condition (*M* = 6.26, SD = 0.68) than in the accuracy-labelled condition (*M* = 2.07, SD = 0.71), Welch t(543.12) = 70.25, *p* < 0.001. This result indicates a difference in average perceived error under the two labels.

The mediators and GAI dependence were measured in the same post-exposure questionnaire. Random assignment concerned the label condition, whereas the mediators were measured rather than independently manipulated. The indirect paths were therefore evaluated as statistical associations, without inferring a temporally established mediator-to-dependence sequence (Maxwell and Cole, 2007).

### 4.4 Statistical analysis

Between-condition summaries and Pearson correlations with GAI dependence were calculated from construct scores. Between-condition differences were examined using two-sided Welch *t* tests, with 95% confidence intervals and Cohen’s *d*. Group-comparison contrasts were expressed as accuracy-labelled minus hallucination-labelled scores. A seven-factor confirmatory factor analysis was estimated using maximum likelihood and compared with alternative models combining trust and controllability, combining the four mediators, or combining all indicators into one factor. Model fit was evaluated using *χ*^2^, CFI, TLI, RMSEA, and SRMR. Internal consistency, composite reliability, average variance extracted, and HTMT ratios were examined, with HTMT confidence intervals obtained from 2,000 condition-stratified bootstrap samples. Detailed HTMT values are provided in Appendix C.

The parallel mediation model included the label condition and all four mediators as predictors of GAI dependence. The label condition was coded 0 for accuracy-labelled and 1 for hallucination-labelled. The joint moderation model additionally included both moderators and all eight mediator-by-moderator interaction terms; continuous predictors were mean-centred. Regression coefficients were reported on the original score scale and evaluated using HC3 heteroskedasticity-consistent standard errors. Indirect associations and indices of moderated mediation were assessed using 95% percentile confidence intervals based on 5,000 condition-stratified bootstrap samples.

Holm’s procedure was applied to the eight interaction tests to adjust for multiple comparisons. The moderation hypotheses were evaluated using two criteria: an interaction coefficient in the hypothesised direction and a Holm-adjusted *p* value below 0.05. Both criteria were required for support. Indices of moderated mediation were reported with pointwise 95% percentile bootstrap confidence intervals as supplementary information about conditional indirect associations. These intervals were not adjusted for multiple comparisons. When the adjusted interaction test and the pointwise bootstrap interval gave different indications, the decision on the moderation hypothesis followed the interaction-based criteria.

Holm’s procedure adjusted the eight interaction tests for multiple comparisons. Simple slopes were examined at the moderator mean and one standard deviation above and below it, holding the other moderator at its mean. VIFs were calculated for the predictors in each regression model. Supplementary models adjusted for education and occupation. Analyses were implemented in Python. Item loadings and reliability and convergent-validity estimates are provided in Appendix B.

## 5 Results

### 5.1 Measurement properties and pooled correlations

The seven-factor model fitted the item data closely: *χ*^2^(539) = 543.17, *p* = 0.442, CFI = 0.9998, TLI = 0.9998, RMSEA = 0.0038 (90% CI [0.0000, 0.0146]), and SRMR = 0.0166. The trust–controllability merged model, the common-mediator model, and the single-factor model had poorer descriptive fit (Table 4). All solutions converged with positive residual variances. Item loadings ranged from 0.879 to 0.997; CR ranged from 0.969 to 0.996, and AVE from 0.858 to 0.989 (Table 5; item loadings in Appendix B).

**Table 4:** Confirmatory measurement-model comparison.

Model	*χ* ^2^	df	CFI	TLI	RMSEA [90% CI]	SRMR
Seven distinct factors	543.17	539	0.9998	0.9998	0.0038 [0.0000, 0.0146]	0.0166
Trust + controllability merged	2047.53	545	0.9403	0.9348	0.0711 [0.0679, 0.0744]	0.0468
Four mediators merged	5603.60	554	0.7994	0.7846	0.1293 [0.1263, 0.1324]	0.0722
All indicators: one factor	15,995.48	560	0.3869	0.3485	0.2249 [0.2219, 0.2279]	0.2200

**Table 5:** Internal consistency and convergent validity.

Construct	Items	Cronbach’s *α*	Loading range	CR	AVE
GAI trust	5	0.981	0.945–0.982	0.987	0.937
Cognitive dissonance	4	0.994	0.987–0.996	0.996	0.985
Perceived controllability	4	0.975	0.960–0.977	0.983	0.936
GAI frustration	3	0.994	0.989–0.997	0.996	0.989
GAI literacy	5	0.984	0.967–0.978	0.989	0.945
Self-efficacy	10	0.981	0.879–0.968	0.984	0.858
GAI dependence	4	0.954	0.916–0.961	0.969	0.887

HTMT ratios ranged from 0.128 to 0.771 (Table 6; detailed values in Appendix C), supporting the assessment of discriminant validity (Henseler et al., 2015). For trust and controllability, HTMT was 0.685, with a bootstrap 95% interval of [0.641, 0.726]. The largest ratio was between dissonance and frustration (0.771, 95% CI [0.738, 0.802]). All point estimates were below 0.85 and their intervals excluded one. This pattern favoured retaining the constructs separately, while the high internal-consistency coefficients (*α* = 0.954–0.994) and strong within-construct item correlations still warranted consideration of item breadth and redundancy (Sijtsma, 2009). A high *α* or a passed attention item alone was not treated as a complete data-quality test.

**Table 6:** Discriminant validity: heterotrait-monotrait ratios (HTMT).

Construct	(1)	(2)	(3)	(4)	(5)	(6)	(7)
GAI trust	–	–	–	–	–	–	–
Cognitive dissonance	0.719	–	–	–	–	–	–
Perceived controllability	0.685	0.671	–	–	–	–	–
GAI frustration	0.693	0.771	0.614	–	–	–	–
GAI literacy	0.298	0.150	0.219	0.128	–	–	–
Self-efficacy	0.367	0.238	0.352	0.238	0.459	–	–
GAI dependence	0.678	0.340	0.531	0.487	0.321	0.402	–

Pooled Pearson correlations with GAI dependence are reported in Table 7; between-condition summaries are presented in Table 8. Dependence was positively associated with trust (*r* = 0.649), controllability (*r* = 0.504), literacy (*r* = 0.307), and self-efficacy (*r* = 0.385), and negatively associated with dissonance (*r* = −0.327) and frustration (*r* = −0.466) (all *p* < 0.001). The four mediators were interrelated, with absolute correlations ranging from 0.590 to 0.751. These pooled correlations describe bivariate relationships across both conditions and need not equal the partial relationships in a model that adjusts for condition and the other mediators.

**Table 7:** Pooled Pearson correlations with GAI dependence.

Variable	*N*	Pearson *r* with GAI dependence	*p*
GAI trust	546	0.649	<0.001
Cognitive dissonance	546	−0.327	<0.001
Perceived controllability	546	0.504	<0.001
GAI frustration	546	−0.466	<0.001
GAI literacy	546	0.307	<0.001
Self-efficacy	546	0.385	<0.001

**Table 8:** Between-condition comparisons of the post-exposure constructs.

Variable	Accuracy-labelled (*M*)	Hallucination-labelled (*M*)	A–H [95% CI]	Welch *t* (df)	*p*	Cohen’s *d*
GAI trust	5.502 (0.833)	4.426 (0.954)	1.076 [0.926, 1.227]	14.036 (534.28)	<0.001	1.201
Cognitive dissonance	2.916 (0.896)	5.143 (0.920)	−2.227 [−2.380, −2.074]	−28.649 (543.60)	<0.001	−2.452
Perceived controllability	4.674	3.710	0.964 [0.819, 1.110]	13.042 (543.99)	<0.001	1.116
GAI frustration	2.505	4.245	−1.740 [−1.887, −1.593]	−23.230 (516.27)	<0.001	−1.988
GAI dependence	3.254	2.935	0.319 [0.176, 0.461]	4.402 (522.12)	<0.001	0.377

Parenthetical values accompanying means are standard deviations. A–H denotes accuracy-labelled minus hallucination-labelled scores.

### 5.2 Between-condition comparisons

Participants in the hallucination-labelled condition reported lower trust and controllability and greater dissonance and frustration than those in the accuracy-labelled condition (all *p* < 0.001; Table 8). The corresponding accuracy-minus-hallucination standardised differences were *d* = 1.201 for trust, −2.452 for dissonance, 1.116 for controllability, and −1.988 for frustration. Dependence scores were also lower in the hallucination-labelled condition (*M* = 2.935 versus 3.254), with a mean difference of 0.319, 95% CI [0.176, 0.461], Welch t(522.12) = 4.402, *p* < 0.001, *d* = 0.377. Table 8 focuses on the five post-exposure constructs. GAI literacy and self-efficacy were measured before exposure and entered as individual-difference moderators, rather than as outcomes of the label condition.

### 5.3 Parallel indirect associations and model diagnostics

The parallel dependence equation explained 52.0% of score variance. With X and all four mediators included, VIFs ranged from 2.035 to 4.100 (Table 9). The VIFs and measurement-level comparisons addressed different sources of uncertainty and were considered separately. In the common adjusted equation, the indirect association was negative through trust (*a b* = −0.568, 95% CI [−0.677, −0.461]), positive through dissonance (0.436, 95% CI [0.282, 0.593]), and negative through controllability (−0.184, 95% CI [−0.260, −0.113]) and frustration (−0.363, 95% CI [−0.496, −0.239]). These directions were consistent with H1-H4 (Table 10).

**Table 9:** Predictor diagnostics.

Predictor	Four mediators only: VIF	X + four mediators: VIF	Full-set tolerance
Label condition	–	2.835	0.353
GAI trust	2.456	2.510	0.398
Cognitive dissonance	2.929	4.100	0.244
Perceived controllability	2.031	2.035	0.491
GAI frustration	2.563	2.884	0.347

**Table 10:** Parallel indirect associations and the adjusted direct coefficient.

Path/term	*a*	*b*	Estimate	Boot SE	95% bootstrap percentile CI
GAI trust	−1.076	0.528	−0.568	0.056	[−0.677, −0.461]
Cognitive dissonance	2.227	0.196	0.436	0.079	[0.282, 0.593]
Perceived controllability	−0.964	0.190	−0.184	0.038	[−0.260, −0.113]
GAI frustration	1.740	−0.209	−0.363	0.064	[−0.496, −0.239]
Total indirect	–	–	−0.679	0.093	[−0.866, −0.499]
Direct	–	–	0.360	0.086	[0.194, 0.527]

*X* = 0 for accuracy-labelled and 1 for hallucination-labelled.

The total indirect association was −0.679 (95% CI [−0.866, −0.499]), and the adjusted direct coefficient was 0.360 (95% bootstrap CI [0.194, 0.527]). Their sum equalled the total hallucination-minus-accuracy contrast of −0.319, which is the same group difference reported in Section 5.2 and Table 8 with the contrast direction reversed. Thus, the positive dissonance pathway offset part of the negative indirect association through the other responses, while the adjusted direct coefficient also pointed in the opposite direction to the total indirect association. This statistical decomposition did not imply an overall increase in dependence or establish a compensatory behavioural process. Removing attention-check failures or never-users, and adjusting for education and occupation, yielded the same signs of the four indirect point estimates (Table 2); these comparisons were not treated as additional bootstrap significance tests.

### 5.4 Moderation and conditional indirect associations

The joint moderation model included both moderators and all eight mediator-by-moderator interaction terms and explained 68.6% of the variance in GAI dependence. The largest VIF was 4.219. Table 11 presents the interaction coefficients, HC3 standard errors, unadjusted and Holm-adjusted *p* values, and pointwise bootstrap intervals for the indices of moderated mediation. Results are organised below by moderator, although all interaction estimates were obtained from the same joint equation.

**Table 11:** Joint second-stage moderation.

Product	*b*	HC3 SE	*p*	Holm *p*	Index	Index 95% percentile CI	*f* ^2^
GAI literacy × GAI trust	−0.042	0.045	0.351	1.000	0.045	[−0.038, 0.145]	0.002
GAI literacy × Cognitive dissonance	0.059	0.032	0.061	0.368	0.132	[−0.007, 0.256]	0.007
GAI literacy × Perceived controllability	−0.125	0.035	<0.001	0.003	0.121	[0.052, 0.186]	0.022
GAI literacy × GAI frustration	−0.038	0.036	0.294	1.000	−0.066	[−0.189, 0.054]	0.002
Self-efficacy × GAI trust	−0.059	0.049	0.228	1.000	0.063	[−0.026, 0.170]	0.004
Self-efficacy × Cognitive dissonance	0.082	0.032	0.011	0.074	0.183	[0.046, 0.316]	0.013
Self-efficacy × Perceived controllability	−0.022	0.041	0.586	1.000	0.021	[−0.054, 0.094]	0.001
Self-efficacy × GAI frustration	0.040	0.039	0.303	1.000	0.070	[−0.052, 0.199]	0.003

Holm-adjusted *p* values refer to the family of eight interaction tests. Support for the moderation hypotheses required both the hypothesised interaction direction and Holm-adjusted *p* < 0.05.

#### 5.4.1 Moderation by GAI literacy

For the trust pathway, the interaction between GAI literacy and trust was not significant (*b* = −0.042, HC3 SE = 0.045, *p* = 0.351; Holm-adjusted *p* = 1.000). The moderated-mediation index was 0.045, with a 95% bootstrap confidence interval of [−0.038, 0.145]. These results did not support the strengthening of the positive trust–dependence relationship predicted by H5a.

The interaction between GAI literacy and cognitive dissonance was also not significant (*b* = 0.059, HC3 SE = 0.032, *p* = 0.061; Holm-adjusted *p* = 0.368). Its positive coefficient did not match the attenuation predicted by H5b. The corresponding moderated-mediation index was 0.132, with a 95% bootstrap confidence interval of [−0.007, 0.256]. H5b was therefore not supported.

In contrast, GAI literacy significantly moderated the relationship between perceived controllability and dependence (b = −0.125, HC3 SE = 0.035, unadjusted *p* < 0.001; Holm-adjusted *p* = 0.003; *f*^2^ = 0.022). The interaction accounted for an additional 0.0069 of explained variance relative to the otherwise identical model omitting this product term. The moderated-mediation index was 0.121, with a 95% bootstrap confidence interval of [0.052, 0.186]. The negative interaction indicated that the positive controllability-dependence relationship became weaker as GAI literacy increased, supporting H5c.

Simple-slope analyses clarified this pattern. With self-efficacy held at its mean, the controllability slope was 0.262 at low literacy, defined as one standard deviation below the mean (95% HC3 CI [0.179, 0.345]), and 0.149 at mean literacy (95% HC3 CI [0.094, 0.205]). At high literacy, one standard deviation above the mean, the slope was 0.037, with an interval including zero (95% HC3 CI [−0.046, 0.120]). The corresponding conditional indirect associations were −0.252, −0.144, and −0.035, with 95% bootstrap intervals of [−0.337, −0.170], [−0.202, −0.089], and [−0.118, 0.043], respectively (Table 12). Thus, the negative indirect association through controllability became less pronounced at higher literacy.

**Table 12:** Controllability slopes and conditional indirect associations by literacy.

Literacy level	Raw GL value	PC slope	Slope 95% HC3 CI	Conditional indirect	Indirect 95% percentile CI
Low (*M* − SD)	4.037	0.262	[0.179, 0.345]	−0.252	[−0.337, −0.170]
Mean	4.935	0.149	[0.094, 0.205]	−0.144	[−0.202, −0.089]
High (*M* + SD)	5.833	0.037	[−0.046, 0.120]	−0.035	[−0.118, 0.043]

For frustration, the literacy interaction was not significant (b = −0.038, HC3 SE = 0.036, *p* = 0.294; Holm-adjusted *p* = 1.000). The moderated-mediation index was −0.066, with a 95% bootstrap confidence interval of [−0.189, 0.054]. These findings did not support H5d. Overall, perceived controllability was the only pathway for which the literacy interaction met both the predicted direction and the multiplicity-adjusted significance criterion.

#### 5.4.2 Moderation by self-efficacy

Self-efficacy did not significantly moderate the trust–dependence relationship (*b* = −0.059, HC3 SE = 0.049, *p* = 0.228; Holm-adjusted *p* = 1.000). The moderated-mediation index was 0.063, with a 95% bootstrap confidence interval of [−0.026, 0.170]. H6a, which predicted a stronger positive relationship at higher self-efficacy, was not supported.

The self-efficacy-dissonance interaction was positive and significant before adjustment (*b* = 0.082, HC3 SE = 0.032, unadjusted *p* = 0.011), but not after Holm adjustment (*p* = 0.074). Its direction was also opposite to the attenuation of a positive relationship predicted by H6b. The moderated-mediation index was 0.183, with a pointwise 95% bootstrap confidence interval of [0.046, 0.316]. Although this interval excluded zero, it was not adjusted for multiple comparisons and did not override the interaction-based decision rule described in Section 4.4. Because the interaction met neither the Holm-adjusted significance criterion nor the hypothesised direction, H6b was not supported.

For perceived controllability, the interaction coefficient was negative but not significant (*b* = −0.022, HC3 SE = 0.041, *p* = 0.586; Holm-adjusted *p* = 1.000). The moderated-mediation index was 0.021, with a 95% bootstrap confidence interval of [−0.054, 0.094]. Thus, H6c was not supported. The frustration interaction was likewise not significant (*b* = 0.040, HC3 SE = 0.039, *p* = 0.303; Holm-adjusted *p* = 1.000), and its moderated-mediation index was 0.070, with a 95% bootstrap confidence interval of [−0.052, 0.199]. H6d was not supported.

Taken together, the results supported H5c but not the remaining seven moderation hypotheses. The supported pattern concerned a weaker positive association between controllability and dependence at higher GAI literacy. None of the self-efficacy interactions satisfied both the directional and multiplicity-adjusted criteria. Table 13 summarises the hypothesis decisions.

**Table 13:** Summary of hypothesis tests.

Hypothesis	Path/interaction	Expected direction	Decision	Interpretation
H1	Label condition → GAI trust → GAI dependence	*a* < 0; *b* > 0; *ab* < 0	Supported	Lower trust in the hallucination-labelled condition was associated with lower dependence, yielding a negative indirect association.
H2	Label condition → Cognitive dissonance → GAI dependence	*a* > 0; *b* > 0; *ab* > 0	Supported	Higher dissonance in the hallucination-labelled condition was positively associated with dependence in the adjusted model, yielding a positive indirect association.
H3	Label condition → Perceived controllability → GAI dependence	*a* < 0; *b* > 0; *ab* < 0	Supported	Lower perceived controllability in the hallucination-labelled condition was associated with lower dependence, yielding a negative indirect association.
H4	Label condition → GAI frustration → GAI dependence	*a* > 0; *b* < 0; *ab* < 0	Supported	Higher frustration in the hallucination-labelled condition was associated with lower dependence in the adjusted model, yielding a negative indirect association.
H5a	GAI literacy × GAI trust → GAI dependence	Strengthening of a positive association; interaction >0	Not supported	The literacy-trust interaction was not significant.
H5b	GAI literacy × Cognitive dissonance → GAI dependence	Attenuation of a positive association; interaction <0	Not supported	The literacy-dissonance interaction was not significant, and its positive point estimate did not match the predicted direction.
H5c	GAI literacy × Perceived controllability → GAI dependence	Attenuation of a positive association; interaction <0	Supported	The positive controllability–dependence association was weaker at higher GAI literacy; the interaction remained significant after Holm adjustment.
H5d	GAI literacy × GAI frustration → GAI dependence	Attenuation of a negative association; interaction >0	Not supported	The literacy-frustration interaction was not significant.
H6a	Self-efficacy × GAI trust → GAI dependence	Strengthening of a positive association; interaction >0	Not supported	The self-efficacy-trust interaction was not significant.
H6b	Self-efficacy × Cognitive dissonance → GAI dependence	Attenuation of a positive association; interaction <0	Not supported	The interaction was positive, contrary to the predicted direction, and did not remain significant after Holm adjustment.
H6c	Self-efficacy × Perceived controllability → GAI dependence	Attenuation of a positive association; interaction <0	Not supported	The self-efficacy–controllability interaction was not significant.
H6d	Self-efficacy × GAI frustration → GAI dependence	Attenuation of a negative association; interaction >0	Not supported	The self-efficacy–frustration interaction was not significant.

## 6 Discussion

This study examined how an identical GAI answer was evaluated when it was identified as hallucinated rather than verified accurate, and how those evaluations related to dependence-related reports. Participants in the hallucination-labelled condition reported lower trust and controllability and higher dissonance and frustration than those in the accuracy-labelled condition. They also reported lower dependence scores. The net result therefore differed from an account in which hallucination information uniformly strengthens dependence.

Trust and controllability concern the credibility and manageability of assistance, whereas dependence concerns difficulties regulating engagement and its consequences. Their positive associations suggest that a tool perceived as reliable and manageable may remain important within users’ coping routines. Frustration was associated with lower dependence in the adjusted model, consistent with the idea that an irritating encounter can diminish the reassurance provided by the tool. These patterns do not make favourable evaluations themselves symptoms of dependence; they locate those evaluations within a broader relationship with GAI.

The positive dissonance pathway contrasted with the three negative indirect associations. This is compatible with conflict about reliance coexisting with a continuing perceived need for the tool. It is not evidence that participants actually used GAI more to restore control or reduce distress. Dissonance was negatively correlated with dependence before adjustment but positively related within the model containing condition and the other responses. The distinction concerns partial versus pooled relationships and should be interpreted alongside the complete predictor set, rather than described as a contradiction or automatically attributed to a compensatory mechanism. Curiosity, verification, and reinterpretation of existing use experiences remain alternative processes to examine with behavioural and temporally separated measures.

This interpretation places the study alongside, rather than in opposition to, recent post-adoption research. Cognitive and emotional routes to GAI reuse and continued engagement are already being investigated (Liu et al., 2026; Satoto et al., 2025). Work on problematic conversational-AI use instead considers regulation difficulties, loneliness, rumination, and mind perception (Hu et al., 2023), while relational studies address attachment and emotional dependence (Laestadius et al., 2024). In a longer randomised study, Fang et al. (2025) distinguished experimental conversation conditions from associations involving voluntary use. These differences in outcome and design matter when comparing a brief labelled encounter with the consequences of sustained chatbot interaction.

The individual-difference results were more selective than the full set of hypotheses anticipated. Greater application-oriented literacy weakened the positive controllability–dependence association, suggesting that users who perceive a broader repertoire of GAI skills may attach less weight to the manageability of a single response. The literacy scale was not an objective test of hallucination detection, so this pattern does not demonstrate superior detection or protection from dependence. No self-efficacy interaction met the adjusted directional criteria. Confidence in using GAI and application proficiency should therefore not be treated as interchangeable buffers, nor should nonsignificant results be recast as evidence of a hidden nonlinear mechanism.

Overall, the cognitive–affective model links evaluations of unreliable output with dependence-related reporting through opposing statistical associations, rather than a single uniform response. I-PACE provides an organising account of these appraisals and person-related resources. Evidence that the processes develop or maintain habitual dependence would require observation of repeated interaction, coping behaviour, and regulation of use over time.

## 7 Implications and limitations

### 7.1 Theoretical implications

First, the study brings symptom-oriented dependence into an area often concerned with trust, task reliance, or continued-use intentions. Retaining this distinction makes it possible to ask whether an unreliable answer changes a local evaluation, alters willingness to delegate, or is associated with difficulties regulating use. The opposing indirect associations show why these outcomes should not be collapsed into a single notion of greater or lesser engagement.

Second, the I-PACE perspective motivates examining person-related resources together with cognitive and affective appraisals. It does not require treating trust as an addiction symptom or treating perceived response controllability as executive self-control. By separating those roles, the proposed model specifies an application of the framework that can be evaluated with temporally ordered evidence rather than claiming to validate its entire account of problematic-use development.

Third, separating credibility, conflict, manageability, and frustration offers a more differentiated account of responses to unreliable GAI. The measurement-model comparisons and HTMT estimates favoured distinguishing these constructs, while the regression diagnostics addressed their joint use as predictors. The literacy–controllability interaction further indicated that one response may carry different implications across users. Together, these findings support examining distinct appraisals without assuming that every proposed path or moderator must be present.

### 7.2 Practical implications

The large differences in evaluations of the same dialogue suggest that accompanying accuracy information is consequential for users’ immediate judgements. Designers and educators should therefore ensure that verification claims are specific and substantiated and that users can inspect the basis for them. Because expert endorsement and accuracy assurance were bundled in the present design, the findings cannot determine which wording or information source is most effective.

For educational and workplace use, evaluating an answer and monitoring one’s pattern of use should be treated as complementary activities. Source checking and correction support address the reliability of a particular output; reflection on unsuccessful attempts to reduce use or interference with responsibilities addresses a different concern. The present study supports distinguishing these targets, but it did not test an intervention that reduces dependence.

Training can combine practical GAI skills with opportunities to verify outputs and decide when independent work is preferable. The literacy–controllability result suggests that the meaning of perceived manageability deserves attention in such training, but it does not show that a literacy intervention reduces dependence. Similarly, the absence of adjusted self-efficacy effects does not warrant classifying individuals as vulnerable solely because of low confidence. Reminders, correction assistance, and adaptive interfaces remain candidates for direct intervention research.

### 7.3 Limitations and future research

Several limitations define the scope of these findings. First, the study operationalised GAI hallucinations through externally supplied accuracy labels attached to an identical static dialogue. It examined responses to an answer identified as hallucinated, without independently varying output accuracy or observing spontaneous error detection. This limits both construct and ecological validity. The manipulation check established a difference in average perceived error, not correct identification by every participant. Expert endorsement appeared only in the accuracy-labelled condition, and no unlabelled condition was included. Future studies could independently vary output accuracy, accuracy information, and expert endorsement, and use interactive tasks to examine how users detect, verify, and respond to initially unlabelled hallucinations.

Second, GAI dependence and the proposed mediators were measured through self-report in the same questionnaire after a single brief exposure. The dependence score reflects participants’ reported tendencies at assessment rather than habitual dependence newly formed during the experiment. The simultaneous measures also leave the temporal ordering of the indirect pathways unresolved, precluding causal conclusions about mediator-to-dependence relationships. Future research should measure baseline dependence, separate cognitive–affective responses and subsequent outcomes over time, and combine repeated assessments with behavioural records of verification, reliance decisions, and attempts to regulate use. Such evidence would help distinguish short-term evaluations from longer-term dependence and evaluate whether compensatory use, information checking, or other processes account for the observed associations.

Third, the sample consisted predominantly of Chinese users and included a high proportion of students, while the stimulus addressed a single unfamiliar technical topic. These characteristics limit generalisation across cultural, occupational, and task contexts. Although participants reported experience with multiple GAI tools, the study did not distinguish platform-specific influences. Future research could recruit more diverse and occupationally balanced samples and compare tasks and platforms that vary in familiarity, perceived reliability, and interaction features. These comparisons would help establish the boundary conditions of the cognitive-affective associations and the observed literacy-controllability moderation pattern.

## Data Availability

The original contributions presented in the study are included in the article/supplementary material, further inquiries can be directed to the corresponding author/s.
