# Supplementary material for: GAI hallucinations and user dependence: cognitive-affective responses and individual differences
Source: Front Psychol. 2026 Oct 9;17:1939367. doi: 10.3389/fpsyg.2026.1939367 (PMC13690793; doi:10.3389/fpsyg.2026.1939367)
Supplement: Supplementary file 1 [file Supplementary_file_1.DOCX]

Supplementary Material

# 1 Appendix A. Stimuli.

Notes: Although this stimulus material has been translated into English here, the original Chinese version was used in the experiment to facilitate participants’ understanding.

# 2 Appendix B. Reliability and validity analysis results.

| Variable | Item | Factor Loadings | Cronbach’s α | CR | AVE |
| --- | --- | --- | --- | --- | --- |
| GAI trust | TRU1 | 0.982 | 0.981 | 0.987 | 0.937 |
|  | TRU2 | 0.974 |  |  |  |
|  | TRU3 | 0.974 |  |  |  |
|  | TRU4 | 0.945 |  |  |  |
|  | TRU5 | 0.965 |  |  |  |
| Cognitive dissonance | CODI1 | 0.996 | 0.994 | 0.996 | 0.985 |
|  | CODI2 | 0.991 |  |  |  |
|  | CODI3 | 0.996 |  |  |  |
|  | CODI4 | 0.987 |  |  |  |
| Perceived controllability | PECO1 | 0.977 | 0.975 | 0.983 | 0.936 |
|  | PECO2 | 0.960 |  |  |  |
|  | PECO3 | 0.961 |  |  |  |
|  | PECO4 | 0.971 |  |  |  |
| GAI frustration | FRU1 | 0.997 | 0.994 | 0.996 | 0.989 |
|  | FRU2 | 0.997 |  |  |  |
|  | FRU3 | 0.989 |  |  |  |
| GAI literacy | LITE1 | 0.968 | 0.984 | 0.989 | 0.945 |
|  | LITE2 | 0.967 |  |  |  |
|  | LITE3 | 0.976 |  |  |  |
|  | LITE4 | 0.978 |  |  |  |
|  | LITE5 | 0.972 |  |  |  |
| Self-efficacy | SELFE1 | 0.936 | 0.981 | 0.984 | 0.858 |
|  | SELFE2 | 0.916 |  |  |  |
|  | SELFE3 | 0.935 |  |  |  |
|  | SELFE4 | 0.939 |  |  |  |
|  | SELFE5 | 0.910 |  |  |  |
|  | SELFE6 | 0.943 |  |  |  |
|  | SELFE7 | 0.968 |  |  |  |
|  | SELFE8 | 0.931 |  |  |  |
|  | SELFE9 | 0.879 |  |  |  |
|  | SELFE10 | 0.901 |  |  |  |
| GAI dependence | DEPE1 | 0.916 | 0.954 | 0.969 | 0.887 |
|  | DEPE2 | 0.952 |  |  |  |
|  | DEPE3 | 0.937 |  |  |  |
|  | DEPE4 | 0.961 |  |  |  |

# 3 Appendix C. HTMT point estimates and percentile intervals.

| Construct pair | HTMT | 95% percentile interval |
| --- | --- | --- |
| Cognitive dissonance – GAI trust | 0.719 | [0.676, 0.757] |
| Perceived controllability – GAI trust | 0.685 | [0.641, 0.726] |
| Perceived controllability – Cognitive dissonance | 0.671 | [0.622, 0.712] |
| GAI frustration – GAI trust | 0.693 | [0.640, 0.738] |
| GAI frustration – Cognitive dissonance | 0.771 | [0.738, 0.802] |
| GAI frustration – Perceived controllability | 0.614 | [0.558, 0.664] |
| GAI literacy – GAI trust | 0.298 | [0.222, 0.378] |
| GL – Cognitive dissonance | 0.15 | [0.066, 0.235] |
| GAI literacy – Perceived controllability | 0.219 | [0.126, 0.307] |
| GAI literacy – GAI frustration | 0.128 | [0.050, 0.209] |
| Self-efficacy – GAI trust | 0.367 | [0.294, 0.437] |
| Self-efficacy – Cognitive dissonance | 0.238 | [0.157, 0.319] |
| Self-efficacy – Perceived controllability | 0.352 | [0.275, 0.421] |
| Self-efficacy – GAI frustration | 0.238 | [0.155, 0.318] |
| Self-efficacy – GAI literacy | 0.459 | [0.392, 0.528] |
| GAI dependence – GAI trust | 0.678 | [0.612, 0.732] |
| GAI dependence – Cognitive dissonance | 0.340 | [0.256, 0.410] |
| GAI dependence – Perceived controllability | 0.531 | [0.452, 0.600] |
| GAI dependence – GAI frustration | 0.487 | [0.413, 0.554] |
| GAI dependence – GAI literacy | 0.321 | [0.233, 0.404] |
| GAI dependence – Self-efficacy | 0.402 | [0.311, 0.485] |
